# Phylogenomic analyses reveal a molecular signature linked to subterranean adaptation in rodents

**DOI:** 10.1186/s12862-015-0564-1

**Published:** 2015-12-18

**Authors:** Kang Du, Liandong Yang, Shunping He

**Affiliations:** Key Laboratory of Aquatic Biodiversity and Conservation of the Chinese Academy of Sciences, Institute of Hydrobiology, Chinese Academy of Sciences, Wuhan, Hubei 430072 China; University of Chinese Academy of Sciences, Beijing, 100049 China

**Keywords:** Subterranean rodents, Convergent evolution, dN/dS, Substitution rate, Positive selection

## Abstract

**Background:**

Genome-wide signatures of convergent evolution are widely expected but rarely revealed in animals. Subterranean rodent genome and transcriptome data produced by next-generation sequencing facilitate the use of phylogenetic methods to infer non-synonymous and synonymous substitution rates within coding regions, which can reveal changes at the molecular level that are correlated with the dramatic shift from a terrestrial to subterranean habitat.

**Results:**

Our study used previously sequenced genome or transcriptome data of two subterranean rodents, the blind mole rat and naked mole rat, and their terrestrial relatives, the mouse and guinea pig, to investigate the genetic basis of rodent subterranean adaptation. An analysis of 4996 orthologous genes revealed that the substitution pace of coding sequences was significantly slower in the blind mole rat than in the mouse, and slower in the naked mole rat than in the guinea pig. The dN/dS ratio was significantly higher in the blind mole rat than in the mouse and in the naked mole rat than in the guinea pig. These patterns are most likely related to the longer generation time and lower effective population size of subterranean rodents caused by subterranean ecological constraints. We also identified some genes and gene ontology (GO) categories that might be candidates for adaptation to subterranean life.

**Conclusions:**

Our study reveals a case of subterranean convergent evolution in rodents that is correlated with change in the pace and mode of molecular evolution observed at the genome scale. We believe that this genomic signature could have also evolved in other cases of subterranean convergence. Additionally, the genes that displayed the most radical changes in their patterns of evolution and their associated GO categories provide a strong basis for further comparative and functional studies, and potentially reveal molecular signatures of adaptation to subterranean life.

**Electronic supplementary material:**

The online version of this article (doi:10.1186/s12862-015-0564-1) contains supplementary material, which is available to authorized users.

## Background

Convergent evolution demonstrates that some aspects of evolution are predictable and repeatable responses to ecological challenges. Molecular evidence of recurrent evolution was observed at the gene level [1–3]; therefore, signature at the genome level is expected as well. Parker et al. analysed genome data of echolocating mammals and suggested that sequence convergence is widespread in genomes [4]. However, this “widespread sequence convergence” pattern was revealed to not be more prevalent than neutral expectations [5, 6]. Additionally, Zou and Zhang suggested that this phenomenon results from prevalent epistasis in protein evolution rather than phenotypic convergence [5]. Foote et al. investigated genome-level marine mammal convergence and suggested that it is rare for molecular convergence to be linked to phenotypic convergence [7]. Consequently, it is interesting to determine if there is a signature of phenotypic convergence at the genome level for unstudied lineages.

Burrowing subterranean rodents spend their lives underground in conditions, such as darkness, low ventilation, hypoxia and high moisture, that are unsuitable for the survival of many animals [8]. By comparison with their surface counterparts, subterranean rodents have evolved many different genetically coded morphological and physiological traits, such as digging forearms or teeth, and modified vestibular organs, eyes and visual processing [9–11]. Furthermore, these animals also have modified demographic parameters, including longer generation time and smaller population size, which might be caused by subterranean ecological constraints that limit opportunities for dispersal and reproductive success [12].

As revealed in a previous study [13], these demographic parameter changes may be associated with nucleotide substitution rate and pattern changes. By analysing morphological traits that may have been positively selected by subterranean life and demography that might be associated with substitution rate and pattern changes, we hypothesized that, contrary to the findings of previous studies [4–7], in this system, a molecular signature would be associated with subterranean convergence that might be identified at a whole genome scale. One approach to determining if there is a molecular signature is through the analysis of the rates of non-synonymous and synonymous substitutions in coding regions [14–16]. The blind mole rat (BMR; *Spalax galili*) and naked mole rat (NMR; *Heterocephalus glaber*) have both adapted to subterranean life, but are members of Spalacinae and Bathyergidae, respectively. Thus, they provide a good model to study the genetic signature of subterranean convergence.

**Fig. 1:**
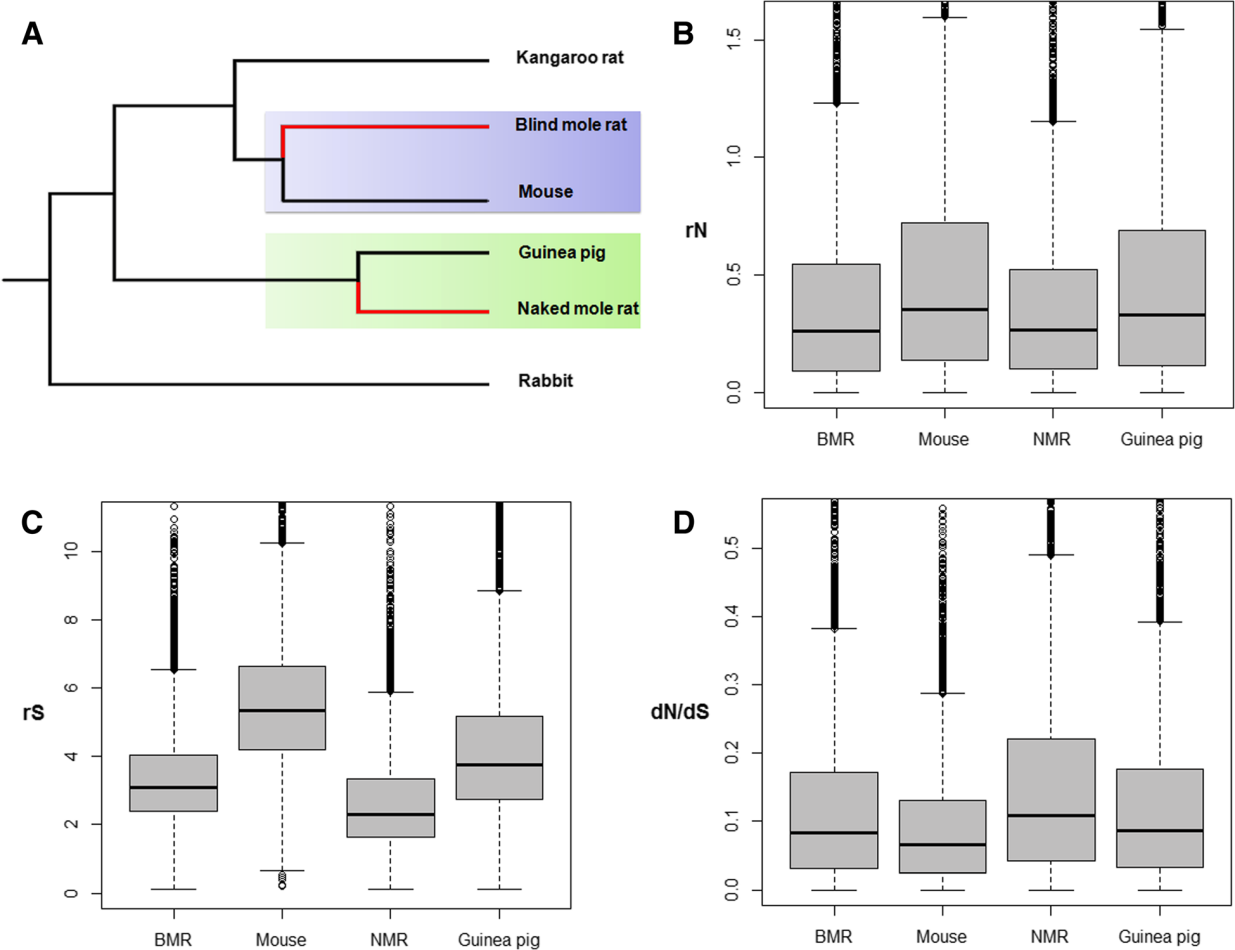
Phylogenetic tree, and substitution rate and pattern for the analysed taxa. **a** A phylogenetic tree that depicts the taxa included in this study. The mouse-related clad is coloured light blue, and the ctenohystrica clad is coloured light green. The red branches represent subterranean rodent lineages. **b**, **c**, **d** Box plots that show the per-site rates of non-synonymous substitutions (rN), per-site rates of synonymous substitutions (rS), and the ratio of non-synonymous to synonymous mutations (dN/dS) of genes for subterranean rodent lineages (the blind mole rat, BMR and naked mole rat, NMR) and their non-subterranean relatives (the mouse and guinea pig, respectively)

In this study, we performed a phylogenomic analysis of the genomes of representative subterranean rodents (the BMR and NMR) and representative non-subterranean relatives (the mouse, *Mus musculus*; and guinea pig, *Cavia porcellus*). We used the mouse and guinea pig as surface references for the BMR and NMR, respectively, when comparing the rate and pattern of genome evolution between subterranean and non-subterranean rodents. The rabbit (*Oryctolagus cuniculus*) was used as the outgroup (Fig. 1a). Our results revealed a significantly slower pace of molecular substitutions and higher dN/dS ratios, which are either directly or indirectly related to subterranean convergence. Additionally, we also identified some genes and gene ontology (GO) categories that have signature of positive or purifying selection in subterranean lineages and therefore might be molecular candidates for subterranean adaptations.

## Results

### Slow substitution rate and high dN/dS ratios associated with subterranean rodent lineages

**Fig. 2:**
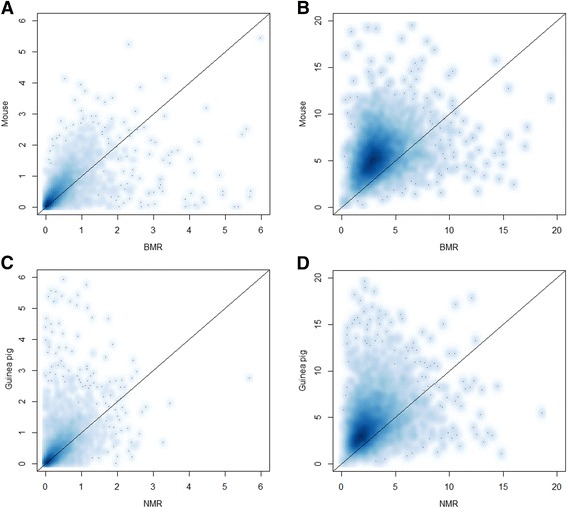
Scatterplots of substitution rate comparison between subterranean rodents and their surface counterparts. **a**, **b** Scatterplots that compare nonsynonymous (rN, **a**) and synonymous (rS, **b**) substitutions between the BMR and mouse. **c**, **d** Scatterplots that compare nonsynonymous (rN, **c**) and synonymous (rS, **d**) substitutions between the NMR and guinea pig

To investigate non-synonymous and synonymous substitution rates related to subterranean convergence in genomic coding regions, we identified 4996 1:1:1:1:1:1 orthologues in genomes of the BMR, NMR, mouse, guinea pig, kangaroo rat and rabbit (Fig. 1a). We determined the per site rates of non-synonymous substitutions (rN) and synonymous substitutions (rS) to evaluate the pace of synonymous and non-synonymous substitutions by dividing dN or dS by the time span of the branches and multiplying by 1000 to yield substitutions/site/year × 10^−9^ [17]. Averaged across all 4996 genes, we found that the BMR had both significantly lower rN and rS than its surface counterpart, the mouse (*p* < 0.0001, Wilcoxon signed-rank tests). The rN and rS were also both significantly lower in the NMR than its surface counterpart, the guinea pig (*p* < 0.0001, Wilcoxon signed-rank tests). This result indicated that pace of molecular evolution is slower in subterranean rat lineages compared with their surface counterpart lineages (Figs. 1b, c and 2; Table 1; Additional file 1: Table S1).

**Table 1:** Mean substitution rates for non-synonymous sites (dN) and synonymous sites (dS), the non-synonymous to synonymous substitution (dN/dS) ratios, and the overall GC content (GC) and GC content at the third codon position (GC3) in the various lineages

	Mean dN	Mean dS	Mean rN	Mean rS	Mean dN/dS	Mean GC	Mean GC3
BMR	0.02	0.163	0.418	3.437	0.137	50.12	55.33
Mouse	0.024	0.265	0.511	5.596	0.106	50.88	57.05
NMR	0.015	0.108	0.371	2.731	0.178	51.01	57.11
Guinea pig	0.021	0.17	0.53	4.294	0.143	50.96	57.01

However, unlike the rN and rS, the average dN/dS ratio was significantly higher in the BMR than that in the mouse (*p* < 0.0001, Wilcoxon signed-rank test) and significantly higher in the NMR than that in the guinea pig (*p* < 2.2e^−16^, Wilcoxon signed-rank test) (Fig. 1d; Table 1; Additional file 1: Table S1), which reveals that non-synonymous substitutions are more prevalent in subterranean rat lineages.

For dN/dS ratio comparison the between BMR and mouse, we further divided genes into two groups, genes with dN/dS both <1 in the BMR and mouse (group 1; 4513 genes), and genes with dN/dS >1 in either lineage (group 2; 27 genes). dN/dS was significantly different in group 1 (0.122 vs. 0.093, *p* < 2.2e^−16^, Wilcoxon signed-rank test), but not in group 2 (2.630 vs. 2.205, *p* = 0.485, Wilcoxon signed-rank test). Comparison between the NMR and guinea pig revealed that, although dN/dS was also significantly different in group 2 (58 genes, 1.995 vs. 1.571, *p* = 0.004, Wilcoxon signed-rank test), there was less different than that observed in group 1 (4482 genes, 0.154 vs. 0.124, *p* < 2.2e^−16^, Wilcoxon signed-rank test). These results indicate that the genomes of subterranean lineages evolved under less stringent negative selection compared with their terrestrial relatives.

### Prevalent GO terms with higher dN/dS ratios in subterranean rodents

GO terms can be seen as representations of functional organization of genomes [18]. To evaluate that if the higher dN/dS ratios of subterranean lineages were associated with particular functional organization, we first identified GO terms that contained at least 10 of the 4996 genes. Then, each GO term was represented by concatenated sequence of its genes. This resulted in 1307 sequences that represented 1307 GO terms. We then used the free ratio model of Codeml (PAML4.8)to calculate dN/dS values for each GO term in each lineage.

**Fig. 3:**
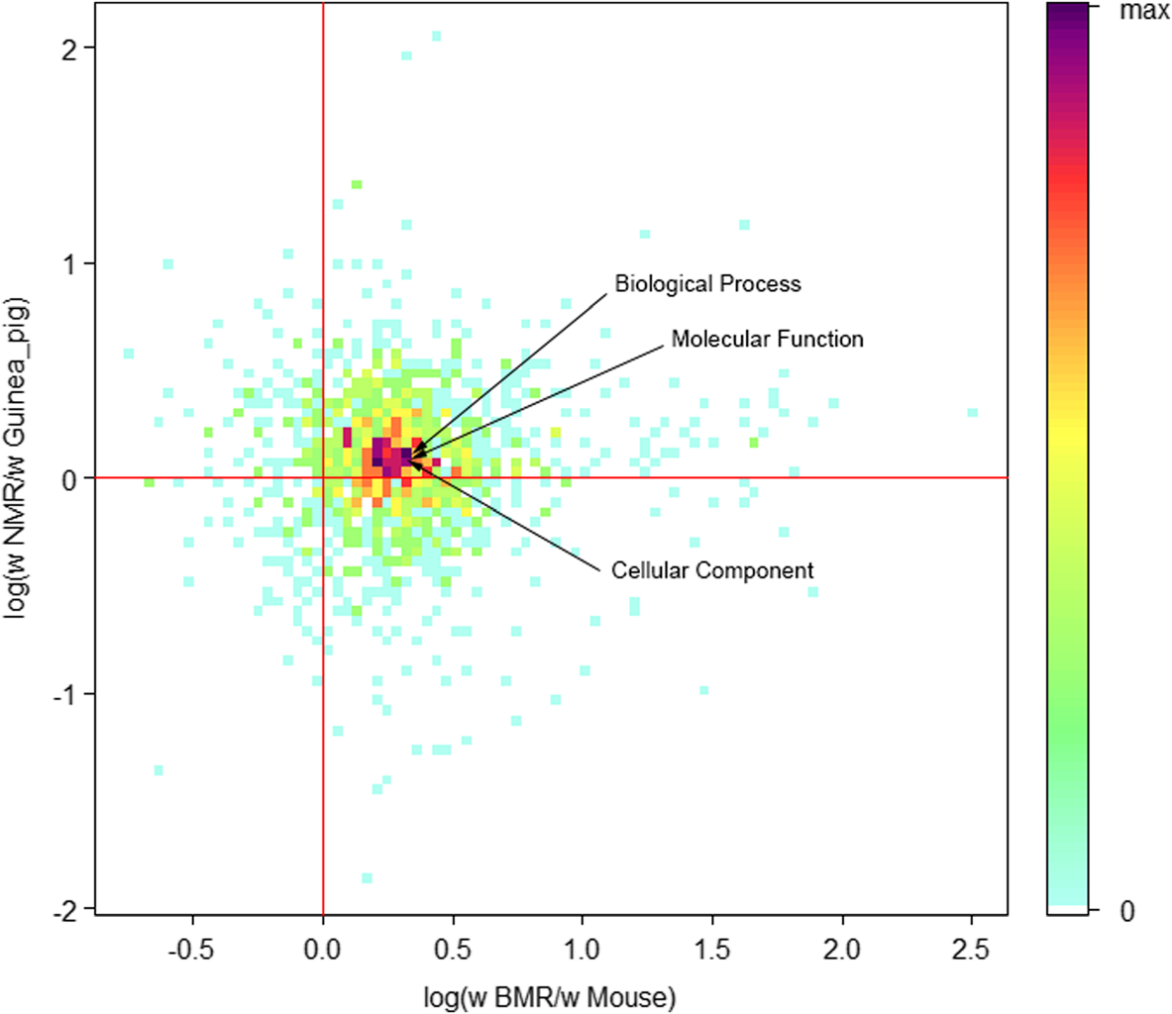
Image scatter plot; the colours indicate the density of the values of log(ω BMR/ω Mouse) and log(ω NMR/ω Guinea pig) for each of the 1307 GO terms identified. The log(ω BMR/ω Mouse) value was obtained by taking the logarithm of the (dN/dS)^BMR^ to (dN/dS)^mouse^ ratio. The log(ω NMR/ω Guinea pig) value was obtained by taking the logarithm of the (dN/dS)^NMR^ to (dN/dS)^GP^ ratio. There were 731 GO terms with log(ω BMR/ω Mouse) and log(ω NMR/ω Guinea pig) values >0; 69 GO terms with log(ω BMR/ω Mouse) and log(ω NMR/ω Guinea pig) values <0; 94 log(ω BMR/ω Mouse) <0 and log(ω NMR/ω Guinea pig) values >0, and 408 log(ω BMR/ω Mouse) values >0 and log(ω NMR/ω Guinea pig) values <0

To compare dN/dS values of GO terms between subterranean lineages and their surface counterparts, for each GO term, we obtained the log(*ω* BMR/*ω* Mouse) value by taking the logarithm of the ratio of (dN/dS)^BMR^ to (dN/dS)^Mouse^ and the log(*ω* NMR/*ω* Guinea pig) values taking the logarithm of the ratio of (dN/dS)^NMR^ to (dN/dS)^Guinea pig^. In total, 731 GO terms (56 %) were identified with values of log(*ω* BMR/*ω* Mouse) and log(*ω* NMR/*ω* Guinea pig) both greater than zero, including the three main GO terms throughout the genome: cellular component, molecular function and biological process (Fig. 3; Additional file 1: Table S2). In addition, the three main GO terms also all passed likelihood-ratio tests (LRTs) in which one-ratio and two-ratio models were compared, and revealed higher dN/dS values in the subterranean lineages (BMR and NMR). These results revealed that, prevalent GO terms (731) were related to the higher dN/dS ratios in subterranean lineages than in their terrestrial relatives, which indicates that the higher dN/dS ratios in subterranean lineages was not influenced by adaptive evolution of some particular functions, but more likely by the less stringent negative selection that impacts the whole genome.

Despite the prevalence of GO terms with higher dN/dS ratios in subterranean lineages, there were still GO terms with lower dN/dS values in the subterranean rodents compared with their surface counterparts, which indicates subterranean-related functional constraints on these GO categories. To identify these GO categories, we processed LRTs for each of the 1307 GO terms. In the LRTs, one-ratio and two-ratio models were compared to search for GO terms with dN/dS values that were lower in the BMR and NMR lineages than in the terrestrial lineages. The *p*-value of LRT was adjusted using the false discovery rate (FDR) method, and 35 GO terms were identified (p_adjust_ <0.01) (Additional file 1: Table S3).

### Influence of BGC on substitution pattern

**Fig. 4:**
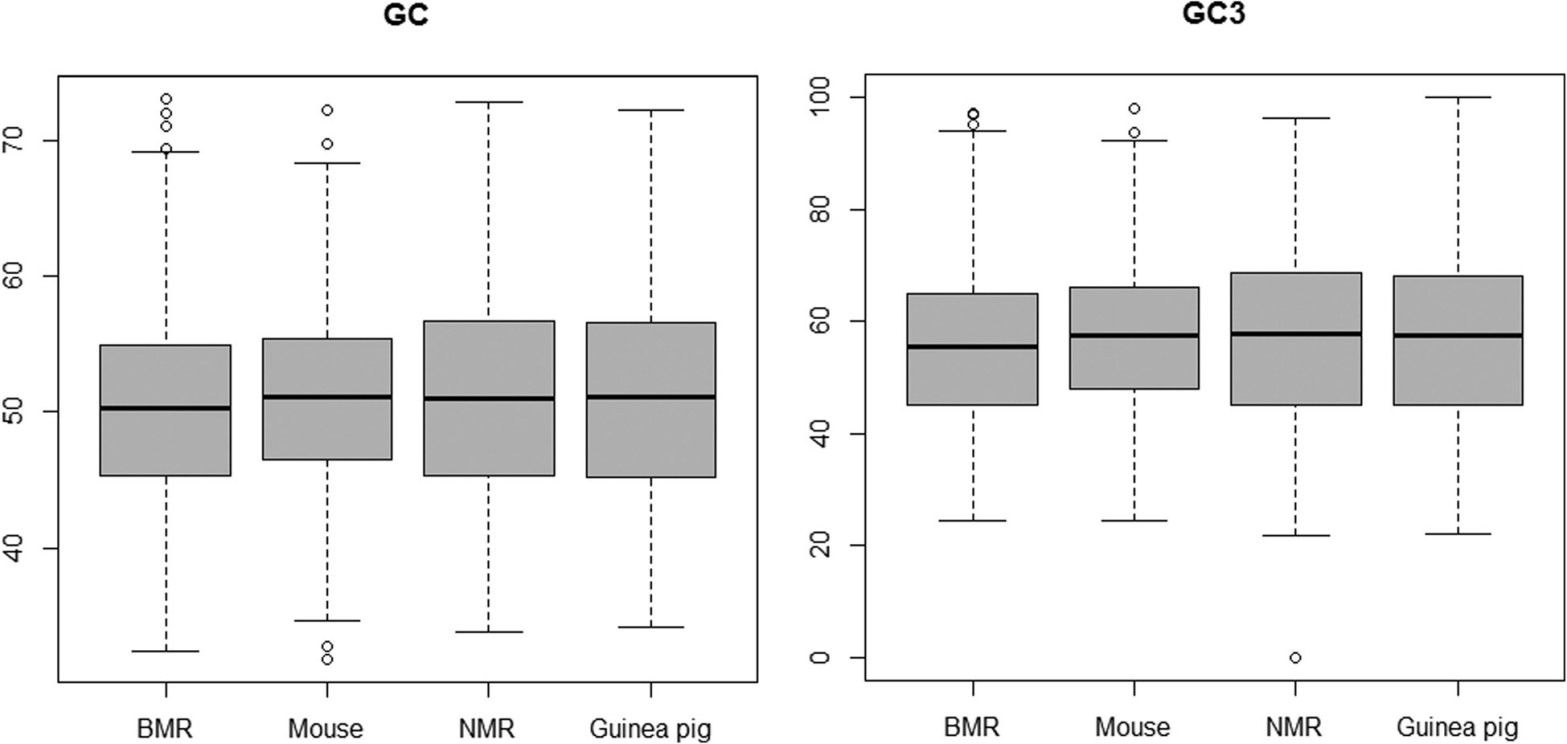
Box plots that show the GC content (GC) and GC content at the third codon position (GC3) for subterranean rodent lineages (the blind mole rat, BMR and naked mole rat, NMR) and their non-subterranean relatives (the mouse and guinea pig)

It was suggested that biased gene conversion (BGC) can produce patterns related to adaptive evolution or relaxed constraints [16, 19, 20]. To investigate a potential role of BGC in determining dN/dS ratio, we estimated GC content evolution across the four lineages using a nonhomogeneous model and revealed a lower GC content (*p* = 7.45e-10, Mann–Whitney *U*-test) in the BMR than in the mouse and almost no difference in GC content between the NMR and guinea pig (Fig. 4, Table 1 and Additional file 1: Table S1). These findings indicate that BGC has a limited effect on dN/dS ratio.

To further examine this phenomenon, we also identified genes with higher dN/dS values in the specific subterranean lineages compared with the rest of the phylogenetic tree, and tested GC content differences of these genes between the subterranean lineage and its surface counterpart. In total, 214 and 237 genes were identified by LRTs in the BMR and NMR, respectively (Additional file 1: Tables S4 and S5, respectively). Average across these genes showed a lower GC content in the BMR than in the mouse (50.97 vs. 51.60, *p* = 8.745e-07, Wilcoxon signed-rank test) and almost no difference in GC content in the NMR and guinea pig (48.07 vs. 48.24, *p* = 0.283, Wilcoxon signed-rank test). This result supported the previous conclusion that there is a limited effect of BGC on dN/dS ratio.

### Positively selected genes

To identify genes that might be involved in subterranean adaptations, we identified positively selected genes (PSGs) that had specific codons influenced by positive selection in only a particular branch by performing LRTs using branch-site models. In total, 451 and 113 PSGs were found in the BMR and NMR, respectively (Additional file 1: Tables S6 and S7, respectively). A subset (*n* = 15) of genes was identified in both groups (Additional file 1: Table S8), including blood vessel-, epithelium- and immune function-related genes, which indicates that these genes might be related to the rodent convergent adaptation to subterranean life.

We also performed GO enrichment analysis for the BMR and NMR PSGs. After correcting for multiple testing, only the BMR showed enrichment of specific GO terms in its PSGs, including axon-, membrane organelle-, protein binding- and helicase activity-related functions (Additional file 1: Table S9).

## Discussion

### Genetic bias associated with subterranean convergence of rodents

We investigated rates of non-synonymous and synonymous substitutions in coding regions of 4996 rodent orthologues, and found a global substitution rate bias associated with subterranean lineages. rN and rS were used to evaluate nucleotide substitution rate, and we found that both are lower in the BMR than in the mouse, and in the NMR than in the guinea pig; this result indicates that coding sequences evolved more slowly in subterranean rodents than their surface counterparts. Many factors that affect mutation and fixation rates are known to influence substitution rates, including metabolic rate, generation time, lifespan and effective population size [17, 21]; generation time was suggested to be the main factor, because species with a short generation time copy their genomes more frequently, thereby accruing more copy errors with time [22–24]. Longevity, age of sexual maturity and (by extension) generation time are, on average, longer in subterranean rodents than in their surface counterparts [12, 25, 26] (Additional file 1: Table S10 and Additional file 2: Fig. S1). These traits might be mainly due to the ecological constraints shared by subterranean lifestyle that limit opportunities for dispersal and reproductive success [12], and potentially contribute to the slower pace of nucleotide substitution in subterranean rodents.

Besides the slower pace of nucleotide substitution, we also found that coding regions of orthologous genes have globally higher dN/dS ratios in subterranean rodents than in their surface counterparts, which indicates that non-synonymous substitutions are more prevalent in subterranean rodents. Moreover, most GO terms were related to higher dN/dS ratios in subterranean lineages, including the three main GO terms throughout the whole genome: “cellular component”, “molecular function” and “biological process”. The fact that the whole genome was impacted might indicate that this phenomenon is linked to neutral forces rather than adaptive evolution.

It was previously suggested that BGC can influence the rate of fixation of particular alleles and result in patterns similar to adaptive evolution or relaxed constraints [16, 19, 20]. However, we found that there was almost no difference in GC content of coding sequences between the NMR and guinea pig, but was lower in the BMR than in the mouse; these findings indicate limited effects of BGC in our system. Although subterranean ecological constraints that limit opportunities for dispersal and reproductive success may lead to the long generation time and small effective population sizes of subterranean rodents [12], an alternative potential cause of the higher dN/dS ratio of subterranean rodents is that there is reduced efficiency of purifying selection due to low effective population sizes under ecological constraints [27].

In summary, subterranean convergence of rodents might lead to ecological constraints that result in longer generation time and smaller effective population size of subterranean rodents. Consequently, less frequent genome replication and reduced efficiency of purifying selection may contribute to the slower pace of nucleotide substitutions and higher dN/dS ratios of subterranean rodent genomes.

### Genes and gene functions important to subterranean rodents

Our analyses revealed 451 and 113 PSGs that have higher dN/dS ratios in the BMR and NMR, respectively. These genes might be candidates for subterranean adaptation of rodents. Moreover, the 35 GO terms with lower dN/dS ratios in subterranean rodents were very likely constrained by selection, and might also be important for rodents to be able to live underground. The underground burrows subterranean rodents live in are dark and unventilated, and the air is low in oxygen but rich in carbon dioxide and ammonia [25, 28, 29]. Such environmental dynamics challenge rodents with hypoxia tolerance, ionic perturbation, hypercapnia and pathogens.

Hypoxia may induce nerve or brain damage [30], carbon dioxide and ammonia may induce acid damage, and blood vessel and epithelium properties may be challenged. In the subset of genes that showed signatures of positive selection in both subterranean rodent lineages, we found genes that might be candidates for this adaptation, such as *Mdga1*, which is involved in neuronal migration [31]; *Megf8*, a modifier of BMP signaling in trigeminal sensory neurons [32]; *Col4a1*, which is involved in small vessel disease [33, 34]; and Zeb1, which is involved in vasculogenic mimicry [35]. Beside these genes, GO terms related to hypoxia and energetic challenges of muscle were also determined to be selection constrained or enriched in PSGs, such as “axon” (GO:0030424), “release of cytochrome c from mitochondria” (GO:0001836), “blood microparticle” (GO:0072562), “positive regulation of smooth muscle cell proliferation” (GO:0048661), and “actin cytoskeleton reorganization” (GO:0031532).

Because of the high moisture, darkness, low ventilation and lack of UV exposure of subterranean rodent burrows, pathogens thrive and challenge subterranean rodents’ immunity [36, 37]. We found immunity-related genes and GO terms that were positively selected or purely constrained in subterranean lineages that might help relieve this pressure, such as *Lyzl6* [38], *Mx2* [39], *Mcam* [40], and “immunological synapse” (GO:0001772).

We also found membrane organelle-related GO terms enriched in PSGs and ionic atmosphere-related GO terms that were constrained in the subterranean rodent lineages. These findings may have been due to ionic perturbation caused by the high content of carbon dioxide or ammonia of the subterranean burrows [41–43].

## Conclusions

In this study, we investigated non-synonymous and synonymous substitution rates in coding regions of certain rodents to determine if there is a global signature for subterranean convergence at the genome level. We found that coding sequences evolved at a slower pace in subterranean rodents than in their surface counterparts, which is potentially due to the longer generation time caused by subterranean ecological constraints. These ecological constraints might also contribute to the lower effective population sizes of subterranean rodents, which may reduce the efficiency of purifying selection; thus, coding sequences of subterranean rodents had globally higher dN/dS ratios. However, we only investigated genome-wide molecular signature in two subterranean rodents; consequently, the limited number of species may lead to potentially speculative results. Additional population genetics analyses of subterranean rodents could help further elucidate these findings.

We also identified blood vessel-, epithelium-, ionic atmosphere- and immune function-related genes and GO terms, which provides insight into the genetic basis of convergent evolution in subterranean environments.

## Methods

### Identification of orthologues

In our study, all data we used were taken from other sources and no animal work was conducted. Protein and CDS sequences from the kangaroo rat (*D. ordii*), mouse (*M. musculus*), guinea pig (*C. porcellus*), NMR (*H. glaber*) and rabbit (*O. cuniculus*) were downloaded from Ensembl version 81 (http://www.ensembl.org). The unigene data for the BMR (*S. galili*) were downloaded from GenBank under the accession number provided by [44]. Peptide sequences were predicted from the unigenes using the Perl script TransDecoder in Trinity. We performed pairwise BLASTP searches between the mouse and other studied species, and assigned genes as 1:1 orthologues using the reciprocal best-hit algorithm. In total, 5511 1:1:1:1:1:1 orthologues were identified.

### Multiple sequence alignment

To obtain reasonable codon-based alignments, we performed multiple sequence alignments for the 5511 identified orthologues using MUSCLE [45] based on the protein sequences. Then, PAL2NAL was used to convert protein sequence alignments into corresponding codon alignments [46]. Alignments that contained sequences with in-frame stop codons were excluded from the data set to eliminate non-homologous alignments. Finally, alignments were trimmed using Gblocks [47] and discarded if shorter than 150 bp (or codons). The use of these filters reduced the dataset to 4996 unique genes.

### Substitution rate estimation

The non-synonymous substitutions per non-synonymous site (dN), synonymous substitutions per synonymous site (dS), and dN/dS ratio (*ω*: the ratio of non-synonymous to synonymous substitutions) for each alignment were estimated using Codeml in PAML 4.8 [48] based on the free-ratio model. Genes with values of dS >1 or S*dS <1 were discarded. The putative orthologues of genes discarded in any one of the six lineages (the BMR, mouse, the ancestral lineage of the BMR and mouse, guinea pig, NMR and the ancestral lineage of the guinea pig and NMR) were also discarded. Each of the five lineages was subsequently represented by 4540 genes.

The rN and rS substitutions were obtained for the BMR, mouse, guinea pig and NMR by dividing dN or dS by the time span of the branches and multiplying by 1000 to yield substitutions/site/year × 10^−9^ [17]. We consulted previous studies to obtain estimates of the divergence dates [25, 49] and assumed blind mole rat/mouse split of 47.4 Ma and a guinea pig/naked mole rat LAC of 39.5 Ma.

Wilcoxon signed-rank tests were used to test whether value substitution rates or dN/dS ratio significantly differed by lineage. All statistical analyses were performed using R.

### Reconstruction of GC content evolution

NhPhyML was used to evaluate changes in GC content for each branch using a non-homogeneous model [50] of sequence evolution for the tree shown in Fig. 1. Both the complete coding region data set and the 3^rd^ codon positions alone were analysed using the default settings.

### Identification of PSGs and genes with higher dN/dS ratios in specific lineages

To identify genes with higher dN/dS ratios in branches of interest, we used the branch model in Codeml [48]. We conducted an LRT between the null model and the alternative model for each gene. The null model postulated that genes evolved at the same dN/dS rate in all branches, whereas the alternative model allowed the ω value to differ between a given branch (foreground branch) and background branches. We also corrected the *p*-value using the FDR method [51] as implemented in R (http://www.R-project.org). Finally, genes with FDR-adjusted *p*-values of <0.05 and higher dN/dS values in the foreground than in the background branches were included.

PSGs [52, 53] were defined as those genes for which only a small number of sites were positively selected in a few lineages [54]. Previously, similar methods barely detected these genes because of the effect of averaging. Here, we applied the branch-site model of Codeml to identify PSGs. An LRT was constructed to compare a model that allows sites to experience positive selection on the foreground branch with a null model, in which sites can evolve neutrally and under purifying selection. The FDR method was applied to correct for multiple testing. Finally, genes with FDR-adjusted *p*-values <0.05 were included.

### GO analysis

We used the TopGO package from Bioconductor (http://www.bioconductor.org) to investigate the enrichment of GO terms; the mouse genome (http://www.ensembl.org) was set as the background. This tool uses a Fisher's exact test and 2 × 2 contingency tables to check for significant over-representation of GO terms in one set compared with another set. All p-values were then adjusted using FDR [51], as implemented in the R package (http://www.R-project.org). GO categories with adjusted *p*-values <0.05 were considered significantly enriched.

## Availability of supporting data

The unigenes of the BMR (*S. galili*) are available at GenBank under the accession numbers JL968997–JL999999 and JO000001–JO020426 [44]. The genomes of other species that were examined in this study are available at Ensembl version 81 (http://www.ensembl.org), references for the specific genome assembly can be found on the More information and statistics page for a species [55]. The life history data in Additional file 1: Table S10 and Additional file 2: Figure S1 were collected from AnAge, the animal ageing and longevity database (http://genomics.senescence.info/species/) [56].

## Additional files

Additional file 1:
**Table S1.** Median values of substitution rates for non-synonymous sites (dN) and synonymous sites (dS), non-synonymous to synonymous substitution (dN/dS) ratios, and overall GC content (GC) and GC content at the third codon position (GC3) in the various lineages. **Table S2.** The 731 GO terms with log(ω BMR/ω Mouse) and log(ω NMR/ω Guinea pig) values higher than zero. **Table S3.** The 35 GO terms with lower dN/dS in subterranean rodent lineages, as indicated by LRTs. **Table S4.** The genes with higher dN/dS values in the BMR compared with other species, as indicated by LRTs. **Table S5.** The genes with higher dN/dS values in the NMR compared with the other species, as indicated by LRTs. **Table S6.** The PSGs in the BMR. **Table S7.** The PSGs in the NMR. **Table S8.** A subset (*n* = 15) of PSGs in the BMR and NMR. **Table S9.** Summary of the over-represented GO terms in subterranean lineages. **Table S10.** Life history data from the AnAge database (http://genomics.senescence.info/species/) for study taxa. (XLS 550 kb) Additional file 2: Figure S1. Life history traits of the study taxa. (**A**) Maximum longevity (yrs). (**B**) Male maturity (d). (**C**) Female maturity (d). (**D**) Litter size multiply by litters per year. (DOC 48 kb)

## Abbreviations

BGC: Biased gene conversion
BMR: Blind mole rat
GO: Gene ontology
LRT: Likelihood-ratio test
NMR: Naked mole rat
PSG: Positively selected gene
