# Supplementary material for: Phylogenomic analyses reveal a molecular signature linked to subterranean adaptation in rodents
Source: BMC Evol Biol. 2015 Dec 18;15:287. doi: 10.1186/s12862-015-0564-1 (PMC4683706; doi:10.1186/s12862-015-0564-1)
Supplement: Additional file 2: Figure S1. — Life history traits of the study taxa. (A) Maximum longevity (yrs). (B) Male maturity (d). (C) Female maturity (d). (D) Litter size multiply by litters per year. (DOC 48 kb) [file 12862_2015_564_MOESM2_ESM.doc]

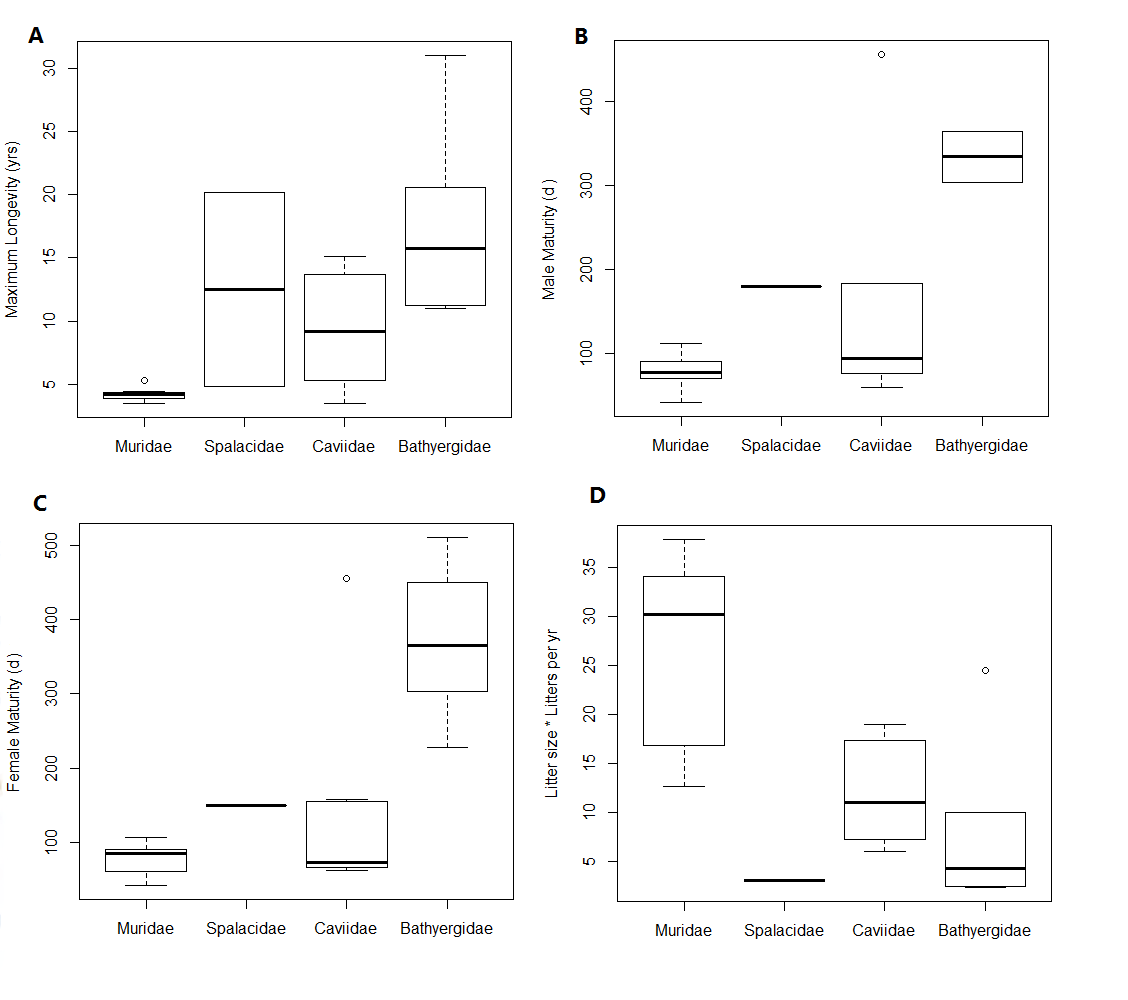


**Figure S1.** Life history traits of the study taxa. (**A**) Maximum longevity (yrs). (**B**) Male maturity (d). (**C**) Female maturity (d). (**D**) Litter size multiply by litters per year.
